# Neotenic Phenotype and Sex Ratios Provide Insight into Developmental Pathways in *Reticulitermes flavipes* (Isoptera: Rhinotermitidae)

**DOI:** 10.3390/insects3020538

**Published:** 2012-06-04

**Authors:** Jian Hu, Brian T. Forschler

**Affiliations:** Department of Entomology, University of Georgia, Athens, GA 30602, USA; E-Mail: hujian@scbg.ac.cn

**Keywords:** Isoptera, life cycle, neotenic differentiation, sex ratio

## Abstract

Several thousand *Reticulitermes flavipes* (Kollar) including worker, nymph, soldier, neotenic and alate castes were collected from three pine logs brought into the laboratory on dates five years apart. The neotenics, all nymphoid, were divided into three groups based on the extent of cuticle pigmentation and termed regular neotenics (RN), black-headed neotenics (BHN) or black neotenics (BN). All castes, from Log A, in 2008, provided a neutral sex ratio except BHN (N = 378) and BN (N = 51) which were exclusively male while the soldiers (N = 466) were female-biased. This information suggests that there is a sex-linked bifurcation along the path for termite development with a male-biased neotenic or female-biased soldier as the choice. In contrast, termites collected in 2004 from Log B provided sex ratios that included a female biased RN (N = 1017), a neutral soldier (N = 258) and male biased BHN (N = 99) and workers (N = 54). Log C, collected in 2009, provided female biased soldiers (N = 32), RNs (N = 18) and BHNs (N = 4) and only male BN (N = 5). Eight laboratory cultures, ranging in age from five to 14 years old, also were sampled and all castes sexed. The census included a 14-year old queen-right colony, an 8-year old polyandrous colony and six colonies provided nymphs and male-biased worker populations. Together these data indicate a flexible caste determination system providing a unique opportunity for a better understanding of the flexible developmental options available in *R. flavipes *that we discuss relative to the literature on *Reticulitermes* ontogeny.

## 1. Introduction

Termites are social insects that undergo paurometabolous development and have morphologically distinct castes [1,2]. They are generally divided into two groups with the higher termites, members of the family Termitidae, representing a diversity of derived species while the remaining families, considered the ancestral lines, are termed the lower termites. The lower termites are known for their developmental plasticity [1]. The various castes and patterns of sex allocation displayed in the lower termites are especially intriguing, but the mechanism underlying aspects of their developmental biology are confused by inconsistencies reported in the literature [3,4,5,6,7,8,9,10,11]. The literature hints at an ontogeny complicated by flexibility. This confusion is exemplified by the fact that there have been at least nine versions of the life cycle proposed for the economically important pest genus *Reticulitermes* [2,6,12,13,14,15,16,17,18]. 

One acknowledged feature of subterranean termite life history is neoteny. Neotenics have long been reported and recognized as occurring in two phenotypes, ergatoids (without wing buds) and nymphoids (with wing buds), arising from worker and nymphal castes respectively [1,2,14,15,19,20]. In this study, we report three nymphoid neotenic phenotypes distinguished by the extent of cuticle pigmentation and present sex ratios of all castes from census of field and laboratory colonies. These observations and data provide valuable insight into the ontological options available to the subterranean termite *Reticulitermes flavipes* (Kollar). We also discuss published life cycles and caste determination/developmental models to illustrate the dynamic nature of *Reticulitermes* biology while suggesting the plasticity shown by this species may contribute to its status as an invasive species.

## 2. Material and Methods

### 2.1. Specimen Collection

**Figure 1:**
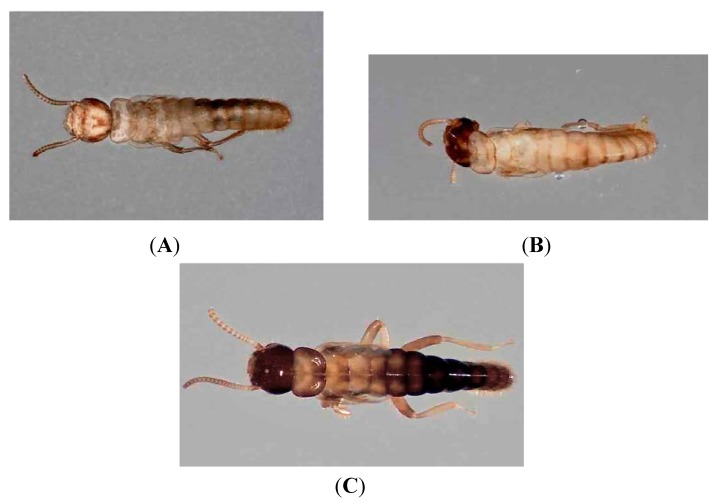
Photographic representation of neotenic phenotype designations based on the extent of cuticle scleritozation. (**A**) Regular neotenic; (**B**) Black-headed neotenic; (**C**) Black neotenic.

*Reticulitermes flavipes* termites were collected at Whitehall Forest in Clarke County Georgia, USA from infested logs, herein designated by capital letters, and extracted as described by Forschler and Townsend [21]. Log A was brought into the laboratory on 5 November 2008 during an unusual, early season swarm of *R. flavipes*. This log provided an alate swarm, indoors, on 9 November 2008 and over the next four weeks produced in excess of 50,000 workers. On 8 December 2008 thousands of neotenics and soldiers began emerging from Log A and this continued for one week. The nymphoid neotenics found in this collection included three phenotypes that were differentiated based on the degree of pigmentation: regular neotenics (RN), black-headed neotenics (BHN) or black neotenics (BN) (Figure 1). RN displayed a light, cream color over the head and body (Figure 1A). BHN had a dark head and a light-colored body (Figure 1B) while BN had a dark head and body (Figure 1C). 

Two more collections of similar neotenic phenotypes were obtained from logs collected at different times from the same locale, designated Log B and Log C. Log B samples were obtained from our voucher collection. The worker, solider and RN’s specimens were collected on 2 April 2004 and the neotenics appeared 4 May 2004. Log C was collected on 15 April 2009 when examination in the field noted BHN. This log was returned to the laboratory and, after several hundred workers and dozens of soliders emerged, neotenics and nymphs appeared between 30 April and 1 May 2009. 

Seven laboratory colonies of *R. flavipes*, herein designated by Roman numerals (I-VII), were cultures started by pairing field-collected alates during the years 2000 to 2004 as described in Grube & Forschler [22]. One laboratory culture (VIII) was initiated by taking a portion of the termites collected from an infested log that produced a primary reproductive pair and over 80,000 workers. Culture VIII was started in 1995 when the aforementioned primary reproductives and 5,000 workers were placed in a 32 × 26 × 9-cm plastic box containing pine wood and maintained as characterized above. Representative samples from all collections reported in this study were stored in 90% EtOH and placed in the voucher collection of the Department of Entomology, University of Georgia, Athens, GA. Neotenics were identified using the criteria of Buchli [15] and the terminology of Thorne [23] used to describe the form.

### 2.2. Sex determination

Sex was determined by the arrangement of sternal plates as described by Zimet and Stuart [24]. The sternal plate character was verified as a correct indication of sex in *R*. *flavipes* workers by dissecting 10–20 of each caste to confirm the presence of ovaries or testis. We examined ≥ 400, randomly selected, termites of each caste for each collection unless otherwise noted (Table 1). Hu and Forschler [25] reported at least 100 termites are required to provide a reasonable estimate of population sex ratios but this number was not obtained for all castes in every collection. Although a sample size smaller than 100 provides a less reliable estimate we report all caste sex ratios with the number sampled to indicate the variability inherent in sampling minority castes and justify conclusions drawn from the data. Allowing for the estimation of error caused by sample size, a sex ratio within 0.50–1.50 was defined as neutral, and otherwise described as biased (Hu and Forschler 2011)[25]. 

**Table 1:** List of castes collected from three logs by caste, including neotenic phenotypes, number examined and resulting sex ratio.

Collection	Caste	♂ (male)	♀ (female)	Sex ratio
Log A	Regular neotenic	306	335	0.91
	Black-headed neotenic	373	5	73.50
	Black neotenic	51	0	exclusively male
	Soldier	157	309	0.51
	Worker	226	174	1.30
	Nymph	155	200	0.78
	Alates	351	271	1.30
	Neotenic +Soldier	887	649	1.37
Log B	Regular neotenic	243	774	0.31
	Black-headed neotenic	94	5	15.40
	Soldier	133	125	1.06
	Worker	34	20	1.70
	Neotenic +Soldier	470	904	0.52
Log C	Regular neotenic	4	14	0.29
	Black-headed neotenic	1	3	0.33
	Black neotenic	5	0	exclusively male
	Soldier	11	21	0.52
	Worker	137	68	2.01
	Nymph	5	13	0.38
	Neotenic +Soldier	21	38	0.55

### 2.3. Results

All neotenics from the Log collections were brachypterous nymphoids and this is the first report of neotenic forms differentiated based on degree of pigmentation believed to be a result of tanning/sclerotization (Figure 1). Variation in the degree of neotenic pigmentation has been mentioned in the literature [20,26] but never quantified leaving us to believe the phenotypes described in this work are considered rare and treated as novelties or not reported. 

The number of termites examined in the sex ratio portion of this study represent a subsample of the animals collected from both Log’s A and B but for different reasons. Log A termites were originally collected for workers to be used in laboratory assays and after neotenics emerged we resampled the Log A culture boxes to obtain worker, nymph, and soldier samples. The alates sexed from Log A were obtained from our voucher collection. There were several thousand members of each caste that emerged from Log A over several weeks and some workers were placed in bioassay, some neotenics sent to colleagues for examination, while others were placed in 90% EtOH and frozen for future study. The remaining 1,070 neotenics, 466 soldiers, 440 workers, 355 nymphs and 622 alates were used for the sex ratios reported herein. The 1,116 neotenics, 258 soldiers and 54 workers from Log B were collected and preserved five years earlier. We do not know whether all the neotenics from that collection were saved, but most certainly not all of the workers were available for this study. Log C provided the smallest number of termites (N = 1000) with a subsample of 205 workers and all neotenics (N = 27), nymphs (N = 18), and soldiers (N = 32) sexed for this report. 

The sex ratio of workers, alates, soldiers, and nymphs in Log A were neutral (1.30, 1.30 and 0.78, respectively), while soldiers (0.51) tended toward female-biased (Table 1). The sex ratio of RN from Log A (0.91) was neutral, whereas the BHN was male-biased (73.50) and the BN were exclusively male (Table 1). The Log B collection provided a female-biased (0.31) sex ratio for the BN, whereas the BHN were strongly male-biased (15.40) (Table 1). The soldier sex ratio from Log B was neutral (1.06) and workers male-biased (1.70). The Log C collection supplied a neutral sex ratio (0.52) for soldiers that tended toward a female-bias, a female-biased sex ratio for the RN (0.29), BHN (0.33), and nymphs (0.38), while the BN were exclusively male and workers male-biased (2.01) (Table 1). 

The castes and respective sex ratios for the laboratory Colonies I-VII are presented in Table 2. Colony I did not have a primary reproductive pair, but contained 62 nymphoid neotenics. The Colony I RN sex ratio was female-biased (0.22), that of the BHN (1.20) and nymphs (0.89) was neutral whereas the soldiers and workers were male-biased (1.83 and 1.74, respectively) (Table 2). Colony II supplied a reproductive caste that was exclusively female RN, a neutral sex ratio for soldiers (1.42), a male-biased worker caste (3.23) and neutral nymphs (0.61) (Table 2). Colony III contained two adult males (kings) and one primary female. The sex ratio for Colony III nymphs was neutral (0.87), however, soldiers and workers were male-biased (1.58 and 1.62, respectively) (Table 2). Colony IV provided neutral nymph (1.00) and soldier (0.72) sex ratios, while the worker caste was male-biased (1.95) (Table 2). Colony V supplied a neutral sex ratio for all castes: soldiers (1.23) and workers (1.48) (Table 2). Colony VI provided a neutral sex ratio for nymphs (1.00), while soldiers and workers (4.43 and 2.48, respectively) were male-biased. There were only two female nymphs in Colony VII with a male-biased sex ratio for soldiers and workers (3.00 and 2.40, respectively) (Table 2). Colony VIII was maintained for 13 years and 11 months in the laboratory and initiated using a field collected reproductive pair providing evidence, assuming the colony growth rates proposed by Grube and Forschler [22], that adult *R. flavipes* adults live for 15–20 years. The nymphs of Colony VIII were exclusively female, soldiers neutral (0.73) and workers male biased (2.70) (Table 2).

### 2.4. Discussion

The serendipitous collection of subterranean termite castes from field populations using the extraction method employed in this study can illuminate biological phenomena that might go unnoticed using other collection methods. Expression of phenotypic plasticity can result from a number of biotic or abiotic factors and the mechanisms involve genetic (transcription/translation) as well as physiological (enzymes/hormones) processes that were beyond the scope of this observational investigation [27]. The fact that we found BHN in the field (Log C) and laboratory cultures (Colony 1) illustrates that the process resulting in pigmented neotenics occurs independently of the conditions implicit in the laboratory collection process, *i.e.*, slow desiccation of the log, lack of temperature fluctuations in addition to disconnecting the captured termites from contact with soil and communication with the entire colony population—assumed to be in the soil or other feeding sites at the time the log was removed from the field. Although the mechanisms involved in producing neotenics need further study, the fact that neotenics are frequently collected combined with these sex ratio data provides insight into *Reticulitermes* developmental pathways. 

**Table 2:** Numbers and sex ratio, by caste, of *R. flavipes* laboratory cultures including date of culture inception and final census.

Collection Paired date	Caste	Date of census	♂ (male)	♀ (female)	Sex ratio
Colony I 2/3/2003	Regular neotenic	10/10/2008	9	41	0.22
	Black-headed neotenic		6	5	1.20
	Nymph		8	9	0.89
	Soldier		11	6	1.83
	Worker		113	65	1.74
Colony II 3/11/2003	Regular neotenic	10/10/2008	0	2	exclusively female
	Nymph		81	132	0.61
	Soldier		17	12	1.42
	Worker		155	48	3.23
Colony III 2/21/2000	Reproductive (K, K, Q)	12/4/2008	2	1	2.00
	Nymph		61	10	0.87
	Soldier		68	43	1.58
	Worker		97	60	1.62
Colony IV 4/21/2003	Reproductive (K, Q)	7/31/2008	1	1	1.00
	Nymph		1	1	1.00
	Soldier		33	46	0.72
	Worker		84	43	1.95
Colony V 4/21/2003	Reproductive (K, Q)	10/23/2008	1	1	1.00
	Soldier		32	26	1.23
	Worker		135	91	1.48
Colony VI 4/21/2003	Reproductive (K, Q)	12/5/2008	1	1	1.00
	Nymph		1	1	1.00
	Soldier		31	7	4.43
	Worker		82	33	2.48
Colony VII 10/6/2004	Reproductive (K, Q)	6/1/2009	1	1	1.00
	Nymph		0	2	exclusively female
	Soldier		9	3	3.00
	Worker		84	35	2.40
Colony VIII 7/25/1995	Reproductive (K, Q)	6/1/2009	1	1	1.00
	Nymph		0	4	exclusively female
	Soldier		19	26	0.73
	Worker		127	47	2.70

## 3. Neotenic Differentiation and Development

Neoteny in *Reticulitermes* has been reported as a common occurrence in the literature for the past 100 years [19,20]. Snyder [28] mentioned that neotenics developed in field populations of *R. flavipes* each spring, in quantities ranging from a few dozen to several hundred; an observation supported by the three swarm-time collections used in the present study. This is corroborated by additional field studies that report a higher proportion of neotenics than primary reproductives [29,30]. The literature is also rife with reports of *Reticulitermes* neotenics found in the presence of a primary reproductive from laboratory cultures [5,19,22,31,32,33]. It is evident that neoteny in *R*. *flavipes* is an independent process that occurs in the presence or absence of primary reproductives. 

The factors that influence the production of neotenics in *Reticulitermes* have never been the topic of directed research although it was generally believed that environmental influences were the primary factor [1,34,35]. A number of recent studies with *R*. *speratus* have illuminated the potential of a genetic component [36,37,38]. The debate between proponents of environmental caste determination (ECD) and genetic caste determination (GCD) as outlined in Lo *et al.* [39] will likely continue as more data is accumulated using molecular techniques [40]. 

An examination of our neotenic data in combination with published reports on castes and caste development can point to avenues of further research. Neotenics have long been reported and recognized as occurring in two phenotypes, nymphoid and ergatoid, with two further, functional distinctions (supplementary, replacement) based on presence or absence of the primary reproductives [1,2,21]. However, our collections provided additional nymphoid phenotypes distinguished by the degree of cuticle pigmentation: RN, BHN, BN (Figure 1). We assume that BHN and BN are terminal stages that received mixed hormonal/abiotic signals as they exhibit the darkened cuticle characteristic of the adult stage but are clearly neotenic because wing development was incomplete and evidenced as 'buds' (did not form into functional wings) while the eyes were, likewise not fully developed (Figure 1). The reason these insects did not follow the 'typical' path to the adult stage but instead transitioned to a neotenic is unknown but the outcome is obvious from looking at the specimens. 

There is ample evidence that neotenics are produced concurrently with alates as a normal development in the life history of certain *R*. *flavipes* populations. This evidence includes the field observations of Snyder [28]; our unpublished data from 80 field collection sites (inspection ports reported in Forschler and Townsend 1996 [21]) sampled four times a year for five years (2000 -2005) that always (N = 6) provided neotenics in collections that also contained alates; and the spring time collection of alates and neotenics from Log’s A, B and C in the present study. The concurrent production of alates and neotenics could be explained by dissemination of a colony-level ‘reproductive’ signal. In response to that signal nymphoid neotenics could occur as a result of timing, abiotic, and/or physiological conditions sending some nymphs to the ‘short-cut’ of neoteny rather than completing the path to adulthood. The Log A, B, and C collections also illustrate that some nymphs switch from the imaginal line to neoteny at a point where a remnant ‘adult’ signal remains - resulting in the gradation of pigmentation (partial sclerotization) evidenced in the BHN and BN.

The difficulty of maintaining healthy *Reticulitermes* cultures illustrates that something is missing in our attempts to mimic the field condition [22,41]. Production of alates and nymphs is rare in *Reticulitermes* cultures [21,32]. In the eight laboratory cultures we examined only three provided significant numbers of nymphs including two that had no primary reproductives. One five-yr-old culture had three primary reproductives one female, two males, and over 100 nymphs providing evidence that nymphs occur in colonies containing the royal pair in addition to highlighting the occurrence of polyandry also reported by Grube and Forschler [21]. Why most laboratory cultures of *Reticulitermes *do not produce a high proportion of nymphs may have something to do with the suggestion that a limited resource results in culling older workers that would normally go to the nymphal stage [21]. As is clear from the work of Lenz *et al.* [30] termites in the field preferentially exploit large food resources adding weight to the hypothesis that resource size and quality have an impact on data obtained from laboratory colony growth and development. Examination of *Reticulitermes* development under varied laboratory conditions must be conducted to elucidate the potential impact of extrinsic factors and without such data laboratory results must be interpreted with circumspection. 

Genetic control of Caste Differentiation (GCD) has recently gained attention with parthenogenesis as the cornerstone of a queen-centric system [36,37,38,39]. That research is unanimous in using a simplified life cycle, assuming that neotenics differentiate (*only*) upon the death of the queen [39]. The GCD life cycle includes a sex-linked inheritance pattern that favors production of alates through inbreeding by worker-derived, ergatoid neotenics [37]. Hayashi *et al.* 2007 [37] used experimental laboratory pairings of various neotenic phenotypes then separated eggs to be reared by workers. They concluded that there is some hormonal control because “in the presence of reproductive individuals, the nymph genotype may be modified into a worker phenotype” [37]. Matsuura *et al.* [28] used natural populations and five microsatellite markers to suggest that neotenics were clones of the queen while workers and “alate nymphs” were almost all produced from sexual reproduction. This body of work is based on the model proposed by Thorne *et al.* [18] that assumes neotenics differentiate *only* as replacement reproductive and has been widely used as the basis for interpretation of microsatellite data [42]. A model that assumes only replacement neoteny, monogyny, an undefined generation time, and direct association of Simple Sequence Repeats (SSR’s) with strict Mendellian inheritance is clearly a good starting point but an oversimplification of the actual system. The fact that there is a genomic component to caste determination is indisputable. Genes are regulated and must be ‘turned on’ to produce mRNA that is translated into proteins, which result in particular phenotypes. The approach of using RNA Interference (RNAi) seems to hold promise for examining the processes underlying the interplay between hormone titers, gene expression, and caste phenotypes [43,44]. 

## 4. Sex Ratios Provide Hints to Developmental Pathways

How does the sex ratio data from our three field populations and eight laboratory cultures/colonies match the literature? The terminology used to describe the various castes confounds comparisons between researchers. Buchli’s [15] 8^th^ pseudergate instar would be called workers using the terminology of Thorne [23]. We will assume that Buchli’s pseudergate is the same as the ‘worker phenotype with a nymph genotype’ mentioned in Hayashi *et al.* [37] or the pseudergate *sensu lato* of Korb and Hartfelder [2]. The confused physical features of partially-pigmented nymphoid neotenics (BN and BHN) would indicate that the hormonal cascade which results in tanning—a rite of passage to the terminal imago stage—is received and (partially) processed by a life stage that doesn’t carry the adult genotype. The Buchli [15] life cycle provides numerous developmental options and can account for the various nymphoid neotenics and caste sex ratios we obtained from the three Log collections in Table 1. 

**Figure 2:**
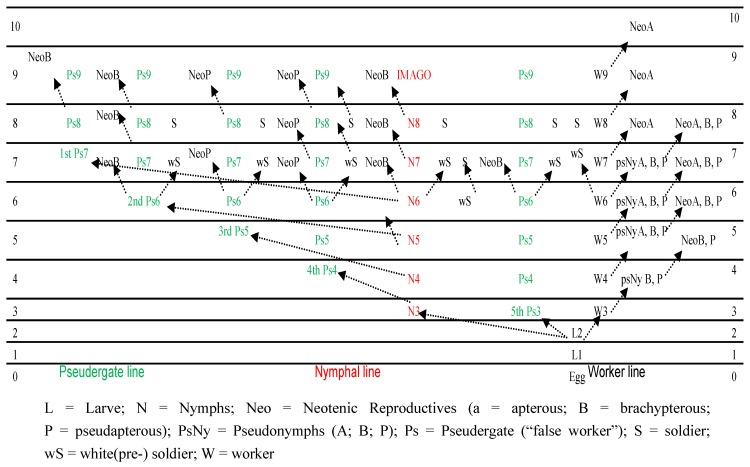
Diagram of Reticulitermes developmental pathways modified from Buchli [15].

The Log A data demonstrates, assuming an equal colony-level sex ratio, a split by soldiers and neotenics from the same developmental path because of the opposite sex ratios in those castes (Table 1). In the Log A collection, the BN and BHN were male-biased, while soldiers were neutral tending to a female-bias. The others castes including workers, alates, RN, and nymphs were obvious neutral. Furthermore, it provided a neutral sex ratio for the combined neotenic and soldier data (Table 1). This information points to a developmental bifurcation with a male-biased neotenic or female-biased soldier as the choice. The life cycle proposed by Buchli [15] has this junction at the 6^th^ nymphal or psuedergate instar while the 7^th^ and 8^th^ instars do not have that option (Figure 2). This system also fits the hypothesis that the BHN and BNs received the signal to become reproductives too late to be alates because the RN, arising from later instars (7^th^ and 8^th^ instars) provided a neutral sex ratio. 

Indicative of the plasticity of *Reticulitermes* development the Log B and C data display different pathways. Logs B and C provided RN ratios that were female biased which is consistent with descriptions in the literature [20,29,33,45,46,47,48,49,50]. Log B had no BN but of the 99 BHN all but five were male. Although this ratio was heavily male biased it does demonstrate that females can also be ‘caught’ in the hormonal confusion assumed to produce these pigmentation-differentiated phenotypes. The fact that the soldier sex ratio from Log B was obviously neutral is contrary to the sex ratios from Log A. The worker sex ratio was however male biased indicating that the bifurcation occurred at a different location in the developmental pathway. The Buchli [15] life cycle again provides that option at the 8^th^ pseudergate instar that is past the soldier pathway (Figure 2). Log C also provided a neutral sex ratio for soldiers (N = 32) that tended to female-bias along with female-biased RN (N = 18), BHN (N = 4) and nymphs (N = 18), while workers (N = 200) and BN (N = 5) were male-biased. These data contrast with the BHN from Log A and Log B that were male biased (Table 1). The three collections (Log A, B & C) provided indications of biased sex ratios in all neotenic phenotypes. Therefore it appears that adult maturation can follow different sex-linked pathways. 

## 5. Sex Ratios and Castes of Laboratory Colonies

*Reticulitermes* appear to have two out of 21 chromosomes that are sex-linked and the presence of a sex-linked developmental pathway should be acknowledged in future research [37,51,52]. The occurrence of one pair of sex-determining chromosomes (XX in females, XY in males) is thought to be the basal sex-determination condition in termites, but fusions or translocations could complicate this simple line of reasoning [53,54]. The life cycle proposed by Vieau [6] indicated a definable morphological differentiation between the worker/soldier and imaginal lines in *Reticulitermes* that is reminiscent of Grasse [14] and Buchli [15]. That split was re-affirmed for *R. speratus *by Hayashi *et al.* [37] who examined 3^rd^ instar offspring after crossing various combinations of nymphoid and ergatoid neotenics and subsequently incubating the eggs in worker-only groups. They found that the sex ratios of nymphs and workers followed a single X-linked locus, dubbed worker (wk), with two alleles, A and B. Their model predicted that offspring in imago-competent colonies would be workers with a neutral sex ratio. Nymph production would only occur should the queen and/or king die and ergatoid neotenics develop [37,39].

Laboratory Colony V was the only group we examined that fit the expected one-locus-allele genetic model proposed by Hayashi *et al.* [37] by having a queen-right colony with a neutral worker sex ratio. Colony V at six years of age contained the primary reproductive pair and only workers and soldiers. Colony V provided a neutral sex ratio for both soldiers (N = 58; sex ratio = 1.23) and workers (N = 226; sex ratio = 1.48) although the worker ratio tended toward male-bias (Table 2). However, the other five queen-right colonies provided male-biased worker populations (Table 2). Nymph production in these same five colonies, however, was as predicted by Hayashi *et al.* [37] because nymphs represented ≤ 1% of the colony population (Table 2). The two ‘queenless’ colonies provided conflicting data regarding nymph production as predicted by the single X-linked genetic model. Colony I contained 61 neotenics including 11 BHN and a population with less than 2% nymphs in contrast to Colony II that had two female RN and a nymphal proportion of 19% (Table 2). Colony II fits the one-locus-allele model for proportional nymphal production but not with the male-biased worker population. Colony I is interesting because BHN were found in a laboratory culture and combined with the low nymph proportions suggests that this culture had some remnant of an ‘adult’ signal—to produce BHNs and low numbers of nymphs despite containing only neotenic reproductives. The variability recorded in the present study with *R. flavipes* may be due to intrinsic biological attributes of that invasive species [55,56,57] compared to other members of the genus.

## 6. Summary

Caste differentiation in *Reticulitermes* is much more plastic than predicted by recently published life cycles [6,18,58,59]. Genetic models predicated on neoteny *only* in the absence of the primary female draw conclusions that are oversimplifications of a complicated biological system. Although recent genetic-centric data sets are arguably valuable in understanding caste differentiation in Reticulitermes, the influence of extrinsic inputs must be part of a holistic approach to understanding the true beauty of this system.

The work of Grassé [14] and Buchli [15] outlined a developmental life history based on observation of morphologically distinct forms. The body of knowledge represented by the literature suggests that this 50-year-old research should be revisited and tested using the powerful tools available in the current century. 

In conclusion, the information gained from this sex ratio study indicates a variety of developmental pathways in subterranean termites that are not consistent with any single model of caste differentiation. Numerous factors have been proposed to affect lower termite caste development including a sex-linked gene [37], juvenile hormone titers [8,9,60], hexamarins [43,44], pheromones [61] and quality/quantity of food resources [62]. The interaction between intrinsic and extrinsic factors must be considered in future studies of what is undoubtedly a complex biological system. 
